# The Role of WO_3_ Nanoparticles on the Properties of Gelatin Films

**DOI:** 10.3390/gels10060354

**Published:** 2024-05-21

**Authors:** Katia Rubini, Arianna Menichetti, Maria Cristina Cassani, Marco Montalti, Adriana Bigi, Elisa Boanini

**Affiliations:** 1 Department of Chemistry “Giacomo Ciamician”, University of Bologna, Via F. Selmi 2, 40126 Bologna, Italy; 2 Department of Industrial Chemistry “Toso Montanari”, University of Bologna, Via P. Gobetti 85, 40129 Bologna, Italy

**Keywords:** tungsten, photochromism, biopolymers, gelatin, nanoparticles, polymer films

## Abstract

Gelatin films are very versatile materials whose properties can be tuned through functionalization with different systems. This work investigates the influence of WO_3_ nanoparticles on the swelling, barrier, mechanical, and photochromic properties of gelatin films. To this purpose, polyvinylpirrolidone (PVP)-stabilized WO_3_ nanoparticles were loaded on gelatin films at two different pH values, namely, 4 and 7. The values of swelling and solubility of functionalized films displayed a reduction of around 50% in comparison to those of pristine, unloaded films. In agreement, WO_3_ nanoparticles provoked a significant decrease in water vapor permeability, whereas the decrease in the values of elastic modulus (from about 2.0 to 0.7 MPa) and stress at break (from about 2.5 to 1.4 MPa) can be ascribed to the discontinuity created by the nanoparticles inside the films. The results of differential scanning calorimetry and X-ray diffraction analysis suggest that interaction of PVP with gelatin reduce gelatin renaturation. No significant differences were found between the samples prepared at pH 4 and 7, whereas crosslinking with glutaraldehyde greatly influenced the properties of gelatin films. Moreover, the incorporation of WO_3_ nanoparticles in gelatin films, especially in the absence of glutaraldehyde, conferred excellent photochromic properties, inducing the appearance of an intense blue color after a few seconds of light irradiation and providing good resistance to several irradiation cycles.

## 1. Introduction

Degradation of native collagen implies rupture of the triple-helix molecules and results in random coil gelatin, one of the most widely used biopolymers. The main sources of gelatin are skin, bones, and cartilage from different animals, usually pigs and cattle but also fish [1]. Gelatin world production is impressive; the annual amount is reported to reach up to 326,000 tons [2], and it is expected to increase significantly in the future [3]. The high interest in this hydrocolloid is due to its unique properties: it is biocompatible, biodegradable, not antigenic, abundant, and relatively cheap, and it can be easily used to form films and coatings [4,5,6]. Its numerous applications span from the biomedical and pharmaceutical fields to food processing and packaging [3,4,7,8,9]. In particular, packaging exploits the ease with which gelatin forms films as well as its oxygen barrier properties [1]. On the other hand, most gelatin applications require bypassing its main drawbacks, including its high solubility in aqueous solutions and consequent poor water vapor barrier properties, as well as its poor mechanical properties [5,10].

Functional and barrier properties can be improved through crosslinking; physical crosslinking includes dehydrothermal treatment, UV, and gamma irradiation, whereas dialdehydes, diisocyanate, carrageenan, and genipin are among the employed chemical agents [5,11,12,13].

Furthermore, it has been reported that the main characteristics of gelatin films can be improved through the incorporation of nanoparticles (NPs) [1]. Several NPs, both metals and metal oxides as well as clay NPs, have been loaded on gelatin films, with consequent improvement of barrier and mechanical properties [1,14,15,16]. However, relatively high concentrations of nanoparticles have been reported to reduce the mechanical strength of nanocomposite films [17]. Furthermore, the presence of NPs has been shown to decrease the light transmission of nanocomposite films as well as their transparency [15,18,19].

WO_3_ is an n-type semiconductor with a variety of applications, which span from gas sensors to smart windows [20], thanks to its peculiar and interesting characteristics, including photochromism [20,21]. In particular, it has been reported that WO_3_ modulates its optical properties under the effect of external light [22] because of an electron–proton double injection [23] that leads to the valence state transformation from W^6+^ to W^5+^ [24]. Recently, WO_3_ NPs have also been reported to display significant antibacterial activity against the Gram-positive *Staphylococcus aureus*, which could be desirable for applications in the biomedical field [25]. Furthermore, WO_3_ NPs were found to induce cell death in osteosarcoma cells while producing no or only minor toxicity in healthy human mesenchymal stem cells [26].

Herein, we explore the influence of WO_3_ nanoparticles on the physicochemical properties of gelatin films. To this end, we loaded gelatin with PVP-stabilized WO_3_ NPs at two different pH values, namely, 4 and 7. The physicochemical properties of pig gelatin films have been previously shown to depend on the pH. Considerable reductions in the triple-helix content, thermal stability, and mechanical properties were observed at pH values higher than 9 and lower than 5 [27].

The effect of the nanoparticles on the main characteristics of gelatin films was also investigated on nanocomposite films crosslinked with glutaraldehyde (GTA) at a concentration of 0.15%, which was previously shown to not cause any cytotoxic reaction [5].

## 2. Results and Discussion

**Figure 1:**
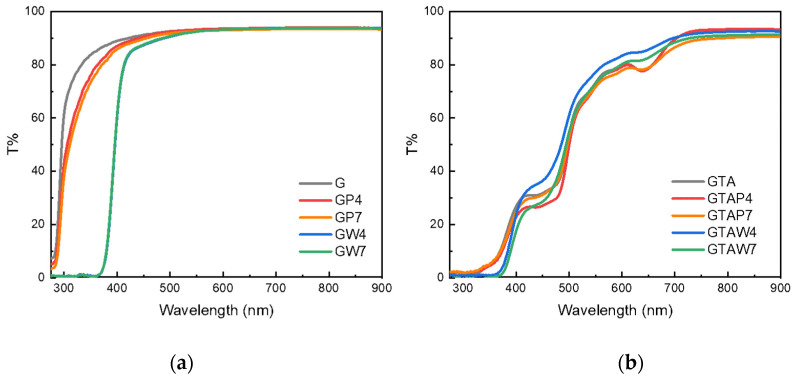
(**a**) Transmittance spectra of uncrosslinked (**a**) and crosslinked (**b**) gelatin films.

Nanocomposite films were prepared from gelatin dissolved in PVP-stabilized WO_3_ sols obtained through an ion exchange technique [26,28]. The hydrodynamic diameter of the nanoparticles measured by means of DLS was below 3 nm, in agreement with previous data [25,26]. Gelatin films, which were colorless in the absence of NPs, assumed a light yellow color when loaded with the metallic oxide nanoparticles (Figure S1). The composite films prepared at different pH values did not exhibit appreciably different intensities, whereas their color became an intense yellow/orange after crosslinking with GTA. The optical properties of films obtained with gelatin (G) and GTA-crosslinked gelatin (GTA) prepared in the presence of PVP at pH 4 (GP4 and GTAP4) and pH 7 (GP7 and GTAP7) and doped with WO_3_ NPs (GW4, GW7, GTAW4, and GTAW7) were characterized through transmittance measurements, as reported in Figure 1.

The transmittance spectra in Figure 1a show that the films obtained in the presence of PVP generated a slight decrease in transmittance in the range of 300–350 nm. Samples GW4 and GW7 showed a strong decrease in transmittance up to about 400 nm, which corresponded to the absorbance of WO_3_ NPs [26] and was in accordance with the appearance of the light yellow color in the films (Figure S1). The transmittance spectra of crosslinked films, as reported in Figure 1b, showed that treatment with GTA led to a decrease in transmittance in all the visible wavelength ranges, mostly between 300 and 500 nm, in accordance with the yellow color of the films. The presence of WO_3_ NPs led to a further decrease in transmittance in the wavelength range of 320–400 nm, as expected.

**Figure 2:**
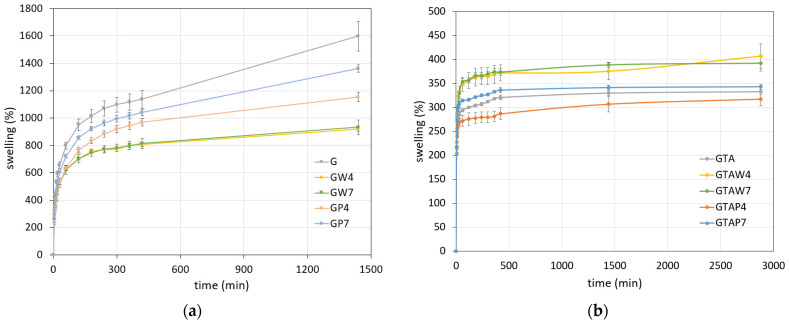
Swelling curves of (**a**) uncrosslinked films and (**b**) crosslinked films. Each value was determined in triplicate.

FEG-SEM morphological investigation revealed the presence of aggregates of nanoparticles on the surfaces of gelatin films containing WO_3_ (Figure S2). Imaging of the single nanoparticles was hindered by their extremely small dimensions and by the presence of the organic matrix, which is greatly sensitive to the electron beam. However, the results of the EDS elemental analysis confirmed the presence of a significant amount of W through the whole surface of the films (Figure S3). Due to the hydrophilic character of gelatin, films undergo remarkable swelling in aqueous solutions. Indeed, the swelling of reference gelatin films was about 1600% after 24 h in PBS. The presence of WO_3_ nanoparticles reduced this value to about 800%, whereas intermediate values were recorded for GP films (Figure 2). Longer times in PBS provoked the dissolution of the films.

Crosslinking with GTA significantly reduced swelling, which assumed mean values between 300 and 400% (Figure 2). Although the data clearly showed that the main influence on this property was crosslinking, GTAW films exhibited slightly higher swelling values than the other series, most likely because the nanoparticles slightly hindered the interaction between gelatin and glutaraldehyde molecules. Moreover, dissolution of the crosslinked films occurred at longer times in comparison to uncrosslinked films.

**Figure 3:**
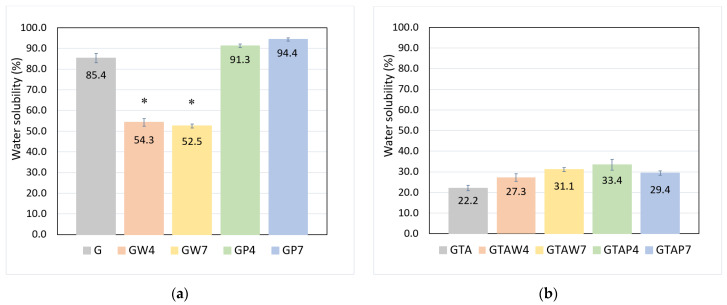
Water solubility of (**a**) uncrosslinked films and (**b**) crosslinked films. Each measurement was performed in 6 replicates. (*) *p* < 0.001 GW4 and GW7 vs. G.

In agreement with the swelling data, the solubility of functionalized films was significantly lower (around 50%) than that of G and GP films (more than 80 and 90%, respectively), as can be seen in Figure 3. Crosslinking not only reduced solubility but also canceled the differences between groups. However, although the difference was not significant, GTAW samples displayed slightly higher values than the other groups, in agreement with the swelling data.

It is known that the presence of nanoparticles increases the thickness of composite films in comparison to that of pristine gelatin films [15,29]. The data reported in Table 1 confirm the greater thickness and reduced water vapor transmission rate (WVTR) of GW films compared to G films but also reveal the important role played by PVP on these properties. On the other hand, the water vapor permeability (WVP) was found to strongly depend on the WO_3_ nanoparticles. In particular, although all the GP and GW samples displayed smaller values than G, the differences were significant only for the GW samples (Table 1). Improved barrier properties in nanocomposite gelatin films were previously ascribed to the creation of zig-zag pathways across water vapors by nanoparticles [1]. No significant differences were found among the crosslinked samples.

The water contact angle is a measure of the hydrophobicity of a surface. Generally speaking, a WCA > 90° is considered characteristic of hydrophobic surfaces, whereas a WCA < 90° is measured for hydrophilic surfaces. As shown in Table 2, all the WCA values were well below the limit of 90°, indicating a hydrophilic character for all the samples, even though the values of PVP-containing samples (GP and GW, GTAP and GTAW) were generally significantly smaller than those of G and GTA, most likely because of the presence of PVP.

The DSC plot of gelatin film displayed an endothermic peak, which allowed two parameters to be obtained: the denaturation temperature, T_D_, and the enthalpy of denaturation, ΔH_D_. Collagen denaturation into gelatin provoked rupture of the triple helices into random coils, while gelling induced a partial renaturation of the triple-helix structure. The endothermic transition associated with the DSC peak was due to the denaturation of the portions of triple helix present in the gelatin films, and T_D_ and ΔH_D_ were related to the thermal stability and the amount of triple-helix structure of the film, respectively [30]. Figure S4 reports the comparison between the DSC plots of gelatin and functionalized samples.

The values of denaturation temperature and denaturation enthalpy of G samples were 93.3 ± 0.3 °C and 22.8 ± 0.4 J/g, respectively (Table 3). GP and GW samples exhibited significantly reduced values of ΔH_D_, most likely due to the presence of PVP, which hindered the renaturation of the triple-helix structure, whereas functionalization did not influence the value of denaturation temperature. Crosslinking slightly increased the thermal stability of the films, as indicated by the increase in denaturation temperature, whereas it caused a decrease in the enthalpy of denaturation, in agreement with previous data [5,31,32]. The remarkable influence of GTA did not show significant differences among the crosslinked samples.

**Figure 4:**
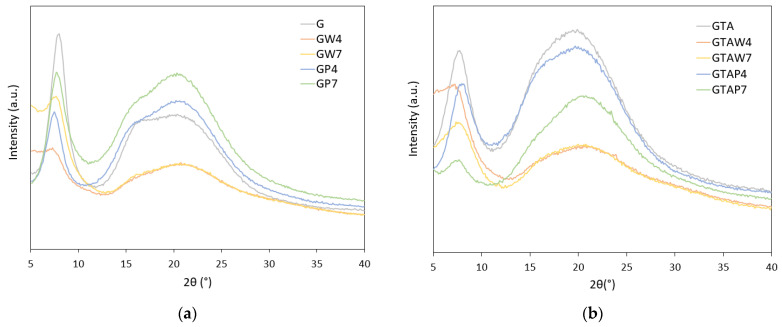
X-ray diffraction patterns of (**a**) uncrosslinked films and (**b**) crosslinked films.

The X-ray diffraction pattern of gelatin provided some insight into its characteristic structure. The XRD pattern of G film, as shown in Figure 4, showed the presence of a peak centered at about 7.5°/2θ, related to the diameter of the portions of triple helices, and a broad halo, centered at about 20°/2θ and related to peptide bonds [33]. The ratio between the relative intensities of these two signals can be considered a measure of the crystallinity of the sample. The higher the relative intensity of the low-angle peak, the more crystalline the sample, as it contains a greater number of ordered portions. The comparison with the profiles from functionalized materials provided evidence of the higher relative intensity of the low-angle peak in the pattern of G films compared to the GP samples, where the presence of the polymer affected the aggregation of the amino acid chains into a triple helix, as observed through the differential scanning analysis. The relative intensity of the peak at about 7.5°/2θ was also low in the patterns of GW samples, but the reduction in the intensity of the whole pattern did not allow a proper evaluation. The comparison of the XRD patterns of crosslinked samples with those of uncrosslinked ones generally revealed a reduction in the relative intensity of the low-angle peak, suggesting a smaller triple-helix content; the aldehydic groups of GTA reacted with the ε-amino groups of lysine and hydroxylysine, thus decreasing renaturation.

**Figure 5:**
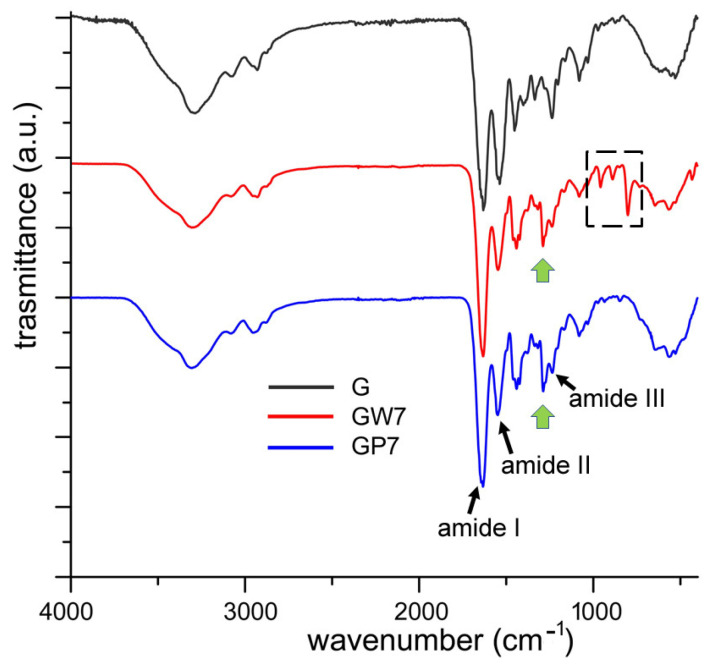
FT-IR spectra of selected uncrosslinked, films collected in ATR mode. Green arrows indicate the intense absorption band at 1290 cm^−1^ due to the C–N bending of the PVP; the dashed line indicates the absorption bands due to the nanoparticles.

Gelatin displayed a FT-IR spectrum characterized by several absorption bands: the main ones fell at 1653 (amide I, C=O stretching), 1550 (amide II, NH bending), and 1238 (amide III) cm^−1^ [34]. These bands were clearly appreciable, although slightly shifted in comparison to those reported by Staroszczyk for fish gelatin, in the spectra of G as well as GTA samples (Figure 5 and Figure S5a). The spectra from GP and GPW films showed the presence of further bands, which cannot be ascribed to gelatin. In particular, the comparison between the spectra of pure PVP and GW7, as reported in Figure S5b, showed that, although several bands of PVP were very close to those of gelatin, the intense absorption band at 1290 cm^−1^ can be ascribed to the C–N bending of the PVP [35]. Moreover, the spectrum from GW7 showed the presence of absorption bands in the region of 1000–800 cm^−1^, which can be attributed to the presence of the nanoparticles. In particular, the intense absorption band at about 810 cm^−1^ was due to the O–W–O stretching mode [36]. The slight shift of the characteristic bands of gelatin in the spectra of the functionalized films (Table S1) suggested a possible interaction with the nanoparticles. No significant differences in the FT-IR spectra were found among the samples prepared at different pH values (GP4 vs. GP7 and GW4 vs. GW7).

The spectra from crosslinked samples displayed minor differences in comparison to those from uncrosslinked films but clearly showed the absorption bands due to the different components (Figure S5a, Table S2).

Functionalization of gelatin films with WO_3_ nanoparticles had a significant impact on their mechanical properties (Table 4). The incorporation of the nanoparticles significantly decreased the values of Young’s modulus and stress at the break of the films. The presence of the nanoparticles introduced discontinuity in the film, and it interfered with the interaction between gelatin chains, thus decreasing stress and elastic modulus [1,15].

As expected, crosslinking with glutaraldehyde drastically reduced the extensibility of pristine gelatin films (strain at break of crosslinked films of about 20% and strain at break of uncrosslinked films of around 150%) and increased the values of the elastic modulus and strain at break (Table 5). Also, in this case, crosslinking reduced the differences among the different samples, although the values of the elastic modulus of GTAW samples were significantly lower than those of GTA, as reported in Table 5.

**Figure 6:**
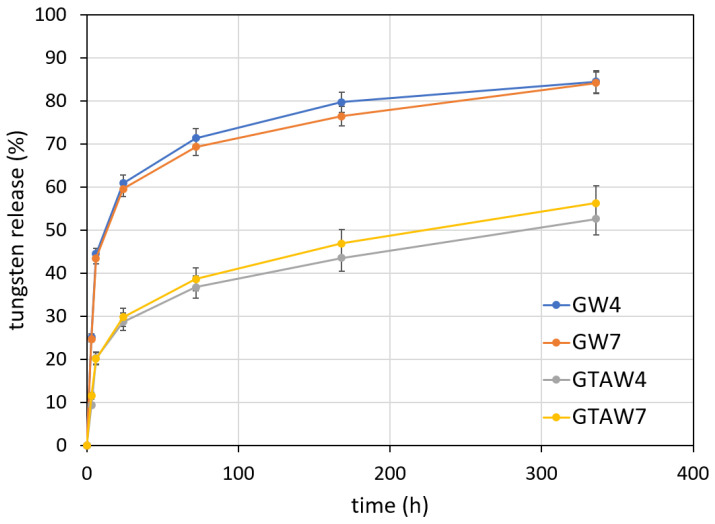
Tungsten cumulative release as a function of soaking time in distilled water.

The W content in the films was 5.0 ± 0.2 %wt (measured with molecular plasma-atomic emission spectroscopy). Figure 6 reports the cumulative release of W in distilled water from the different samples. About 70% of the initial W content was released from uncrosslinked samples after 3 days, with no appreciable difference as a function of pH. Longer exposure to distilled water provoked a much slower release, so its cumulative value reached about 80% at 14 days. Crosslinking significantly reduced tungsten release, which was less than 40% after 3 days and less than 60% after 14 days. Most likely, the different behavior of crosslinked and uncrosslinked samples can be attributed to the different solubility of the films: gelatin is highly soluble in water, and glutaraldehyde crosslinking remarkably reduces the hydration of the films.

**Figure 7:**
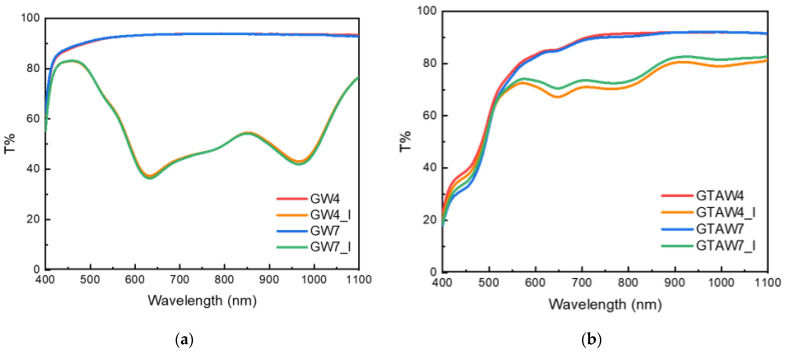
Transmittance spectra of gelatin films modified by WO_3_ NPs before and after irradiation. (**a**) GW4 and GW7 spectra before and after irradiation (GW4_I and GW7_I). (**b**) GTAW4 and GTAW7 spectra before and after irradiation (GTAW4_I and GTAW7_I).

The photochromic properties of gelatin films functionalized with WO_3_ NPs were evaluated by irradiating the film surface with an LED at 365 nm, a wavelength that matches the absorbance of WO_3_ NPs [26,37]. After even a few seconds of irradiation, a blue color started to appear, and its intensity increased with irradiation time. The color change was already very visible in all the films after 20 s of irradiation (Figure S6). Samples GW4 and GW7 became blue, whereas the color of GTAW4 and GTAW7 became greenish due to the yellow color of the films. The transmittance spectra of WO_3_-loaded gelatin films after irradiation (20 s) are reported in Figure 7.

Figure 7a shows that the photochromic reaction in GW4 and GW7 led to the formation of a broad band between 500 and 1100 nm, with characteristic peaks at 610 and 980 nm and a shoulder at 800 nm, in agreement with the typical absorbance peaks present after the photochromic process of WO_3_ NPs [23]. The transmittance of the peaks at 610 and 980 nm was about 35% after only 20 s of irradiation. Indeed, the photochromic process was very fast: the transmittance already dropped to almost zero after 4 min of irradiation at 365 nm (Figure S7a). The photochromic process is related to the transformation of W^6+^ to W^5+^ due to the electron–proton double injection [23,38]. Thus, proton donors enhance the efficiency of the photochromic process, and the presence of hydrogen bonding favors proton diffusion [39]. In this system, this role can be played by the hydroxyl groups provided by PVA [22]. Moreover, the hydroxyl groups present in the gelatin structure can further contribute to photochromic efficiency.

GTAW4 and GTAW7 exhibited different photochromic behavior: their spectra showed the characteristic band, but the transmittance obtained was almost 70%. Indeed, the transmittance of GW4 and GW7 reached almost zero after four minutes of irradiation, while in GTAW4 and GTAW7, the same effect was achieved after one hour of irradiation (Figure S7). The lower photochromic efficiency of the crosslinked samples was probably due to the yellow color given by GTA. As can be observed in Figure 1, the absorbance of GTA was also present in the range of WO_3_ absorbance (300–400 nm). Thus, less irradiation light was absorbed by WO_3_ NPs in GTAW4 and GTAW7 compared to GW4 and GW7. The characteristic peaks at 610, 800, and 980 nm were also present in GTAW4 and GTAW7, despite the intensity ratios being slightly different. This could be due to the band structure already present in the GTA–gelatin films (Figure 1).

**Figure 8:**
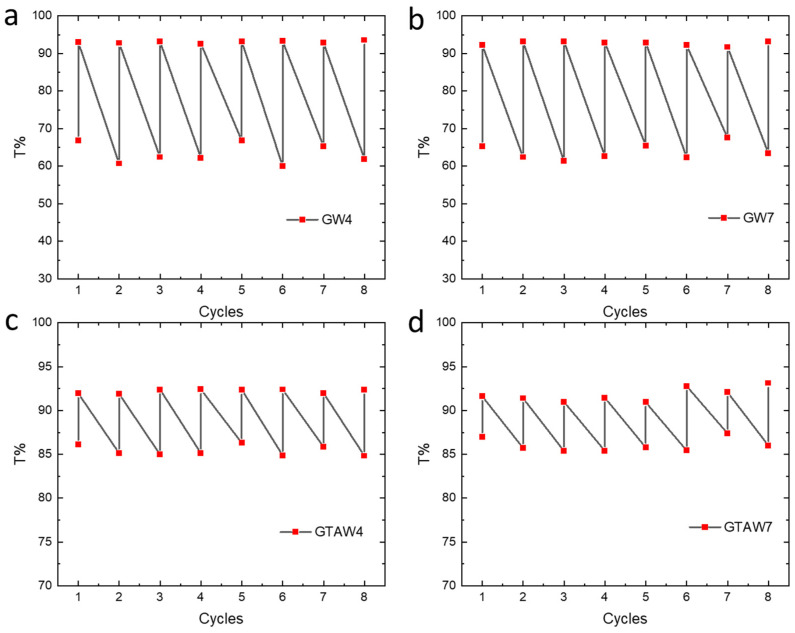
Transmittance variation during 8 irradiation cycles of gelatin films modified by WO_3_ NPs: (**a**) GW4, (**b**) GW7, (**c**) GTAW4, (**d**) GTAW7. Photochromic behavior was monitored by performing transmittance spectra before and after irradiation and considering the peak minimum at 983 nm to identify the photochromic process.

All the samples irradiated for 20 s showed a complete reverse photochromic reaction after exposure to air for three days. This slow reversibility process was not accelerated either by light irradiation (irradiation performed at 660 and 850 nm) or by thermal heating (performed at 60, 80, and 100 °C). The reverse photochromic reaction was due to the oxidation of W^5+^ to W^6+^ by oxygen and can be slowed by the stabilization of W^5+^, given its interaction with groups such as COOH and OH [40]. According to this consideration, in our system, the hydroxyl groups that favored photochromism could also have a role in the slow bleaching. The bleaching effect of gelatin and GTA–gelatin samples was evaluated by irradiating the samples under the same conditions. Although the crosslinked samples had less efficient photochromism, the bleaching time was the same as for the gelatin samples. Thus, it can be deduced that the bleaching of GTA–gelatin samples is less preferred than that of gelatin samples. GTA crosslinking probably further stabilizes W^5+^, slowing the reverse photochromism. Photochromic behavior was also monitored under eight irradiation cycles for all the samples (Figure 8). Each irradiation cycle was performed by irradiating the films for ten seconds at 365 nm and monitored by transmittance spectroscopy; irradiation cycles were performed every two days, allowing the complete reverse reaction before each cycle.

As shown in Figure 8, the photochromic process was not affected by multiple irradiation cycles. When irradiated, the film photochromic response led to almost the same transmittance values for all the cycles, showing an average transmittance of 63 ± 3% and 63 ± 2% for GW4 and GW7, respectively, and 85 ± 1% and 86 ± 1% for GTAW4 and GTAW7, respectively. As expected, the transmittance values obtained for the crosslinked gelatin films were higher due to the less efficient photochromic effect as a result of the presence of GTA, while no differences were reported when comparing samples prepared at pH 4 and 7. Moreover, all the films presented a complete reversible process after irradiation, leading to a total disappearance of the blue color after two days of air exposure of the samples for every irradiation cycle. These results denote a very good resistance of the photochromic properties of the gelatin films to several cycles of light irradiation. In particular, the high efficiency, resistance, and stability of photochromic materials, coupled with control over reversibility, are pivotal for their application in optical memory devices, one of the fields in which the photochromic effect has been most investigated [41,42,43]. According to this, after additional investigation on the bleaching process, our WO_3_-based gelatin photochromic material, which shows efficient and stable photochromism, could find an interesting application in optical information storage.

## 3. Conclusions

The results of this work show that the properties of gelatin films can be modified through functionalization with WO_3_ nanoparticles. Loading of PVP-stabilized WO_3_ NPs reduces swelling, solubility, and water vapor permeability while maintaining the hydrophilicity of gelatin films. On the other hand, the decrease in denaturation enthalpy and relative intensity of the low-angle X-ray reflection observed in GP and GW samples are mostly due to the presence of PVP, which hinders the renaturation process of triple-helix structures. WO_3_ NPs provoke a significant reduction in the values of elastic modulus and stress at break, most likely because they introduce zones of discontinuity in gelatin films. The above-mentioned properties, as well as the cumulative release of W (about 80% after 14 days in distilled water), were not significantly influenced by the different tested pH values, whereas they were significantly affected by crosslinking with glutaraldehyde, which showed the differences among the samples.

The WO_3_ NP photochromic reaction in gelatin films is very fast (from a few seconds to a few minutes of irradiation at 365 nm), while the bleaching process occurs after some days. Both the quickness of the photochromic reaction and the slowness of the bleaching can be explained by the presence of hydroxyl groups present in both PVA and the gelatin structure. The presence of GTA in gelatin films makes the photochromic reaction less efficient and the bleaching process slower. Moreover, in all the samples, no decrease in performance under several irradiation cycles was observed, denoting an excellent resistance of the WO_3_ NPs in the films. In summary, WO_3_–gelatin films represent an interesting system with very efficient and stable photochromic properties. Further control over the reversibility of the photochromic effect could lead to their application as optical information storage devices.

## 4. Materials and Methods

### 4.1. Synthesis of WO_3_ Nanoparticles

The synthesis of tungsten oxide nanoparticles was carried out as previously reported [25,26]. Briefly, 200 mL of a solution containing 0.005 mol of Na_2_WO_4_∙2H_2_O (Sigma Aldrich, St. Louis, MO, USA) was passed through 51.0 g of Amberlite^®^ IR120, a strongly acidic cation exchange resin, and dropped to a flask containing 4 g of solid polyvinylpyrrolidone (PVP-10, av. MW: 10,000, Merck, Darmstadt, Germany). The solution was kept for 4 h at 100 °C under stirring, yielding a clear yellow color. Afterwards, the pH was adjusted to 4 or 7 with diluted NH_4_OH, and the resulting solution was used for the preparation of gelatin films containing WO_3_ nanoparticles. The nanoparticle hydrodynamic diameter was measured using dynamic light scattering (DLS) (ZetasizerNano; Malvern Instruments Ltd., Malvern, UK).

### 4.2. Film Preparation

A solution of 5 g of gelatin from pig skin (280 Bloom, Italgelatine S.p.A., Cuneo, Italy) in 100 mL of water was obtained at 40 °C under stirring (450 rpm); afterwards, 7.4 mL of gelatin solution was placed into Petri dishes (diameter = 6 cm), and films were formed at room temperature after solvent evaporation under a laminar flow hood. These films were used as reference materials and labeled as G.

Films containing WO_3_ nanoparticles were prepared as reported for the preparation of pure gelatin films but by dissolving 5 g of gelatin from pig skin in 100 mL of a yellow solution containing WO_3_ nanoparticles. Samples were labeled as GW4 or GW7, depending on the pH of the WO_3_-containing solution (pH 4 or 7, respectively).

Because the solutions containing WO_3_ nanoparticles also contained a significant amount of polyvinylpyrrolidone, gelatin films were also prepared in PVP-10. To this end, 4 g of PVP-10 was dissolved in 200 mL of distilled water and the pH adjusted to 4 or 7; afterwards, additional reference films were prepared following the same procedure reported above but by dissolving gelatin in PVP-10 solutions. Samples were labeled as GP4 or GP7, depending on the pH of the PVP-containing solution (pH 4 or 7, respectively).

Crosslinked films were prepared following the above procedure. In addition, after air drying, 7.4 mL of a 0.1 M phosphate buffer solution (PBS, pH 7.4) containing glutaraldehyde (GTA) 0.15 %wt was poured onto each film in a Petri dish. After 24 h, the solution was removed, and the films were carefully washed with 1 M glycine solution and dried under a laminar flow hood. Crosslinked films were labeled as GTA (prepared in water), GTAW4 and GTAW7 (prepared using the WO_3_-containing solution—pH 4 and 7, respectively), and GTAP4 and GTAP7 (prepared using the PVP-containing solution—pH 4 or 7, respectively).

### 4.3. Film Characterization

Film morphology was observed using a high-resolution scanning electron microscope (FEG-SEM, Zeiss Leo-1530, Oberkochen, Germany) operating at 5 kV equipped with an energy-dispersive X-ray spectrometry system (EDS, Oxford Instruments, Abingdon, Oxfordshire, England) for the elemental composition analysis. The substrates had previously been made conductive by sputtering with gold.

For swelling tests, films were cut into squares (1 × 1 cm). Each specimen was soaked in 15 mL of 0.1 M PBS at room temperature. At selected time points, wet samples were gently wiped with filter paper to remove excess water. The amount of absorbed water was calculated as follows:

W(%) = [(W_w_ − W_d_)/W_d_]·100

where W_w_ and W_d_ are the weights of the wet and dry specimens. Each measurement was performed in triplicate.

For film solubility determination [44], a portion (2 × 2 cm) of each film was soaked in 15 mL of distilled water under gentle stirring (65 rpm) at room temperature. After 15 h of soaking, each specimen was filtered in order to recover the remainder of the undissolved film on filter paper, which was dried at 105 °C for 24 h. The film solubility was calculated as follows:

FS (%) = [(W_0_ − W_f_)/W_0_]·100

where W_0_ is the initial weight of the film expressed as dry matter, and W_f_ is the weight of the desiccated, undissolved rest of the film. Each measurement was performed six times.

Water vapor permeability (WVP) was evaluated using the modified ASTM E96-93 method [45,46]. Briefly, glass vials containing 2 g of anhydrous CaCl_2_ were closed by means of circles of films (diameter = 2 cm) that were glued with silicon on the opening. Vials were placed in a glass desiccator containing a saturated Mg(NO_3_)_2_∙6H_2_O solution (75% RH at 25 °C). The vials were weighted daily for up to 12 days. The WVP was calculated as follows:

WVP (g s^−1^ m^−1^ Pa^−1^) = (ΔW/Δt)·[χ/(A·ΔP)]

where ΔW/Δt is the amount of water gained per unit time of transfer, χ is the film thickness, A is the exposed area of the samples, and ΔP is the water vapor pressure difference between the two sides of the film (1670 Pa at 25 °C, table value). Film thickness was measured with a digital micrometer (Mitutoyo, Kawasaki City, Japan). Each measurement was performed in 6 replicates.

The contact angle was measured on film surfaces using a KSV CAM-101 instrument. Images of deionized water drops were recorded in a time range of 0–10 s. Each measurement was performed in triplicate.

Calorimetric measurements (DSC) were carried out on dry samples at 5 °C min^−1^ in the temperature range of 40–110 °C (PerkinElmer Pyris Diamond differential scanning calorimeter). The denaturation temperature was determined as the value of the corresponding endothermic peak. Denaturation enthalpy was calculated considering the sample weight. Each measurement was performed in triplicate.

X-ray diffraction analysis was carried out in the angular range of 5–40°/2θ (step size 0.1° and time/step 200 s) by means of a Panalytical XCelerator powder diffractometer (λ = 1.5406 Å, 40 mA, and 40 kV).

Fourier transform infrared spectra were recorded using a Bruker ALPHA FT-IR spectrometer in ATR mode. Infrared spectra were acquired at room temperature from 4000 to 400 cm^−1^ with a resolution of 4 cm^−1^.

For mechanical tests, films were cut into stripes (3 mm × 30 mm) after soaking in 0.1 M PBS, pH 7.4, for 1 min. Thickness was measured for every stripe (values around 0.12 mm) using a Leitz SMLUX-POL microscope (Leica Microsystems, Milano, Italy). Stress–strain curves of strip-shaped films were recorded with a crosshead speed of 5 mm min^−1^ (INSTRON Testing Machine 4465, Norwood, MA, USA). The Young’s modulus (E), the stress at break (σ_b_), and the strain at break (ε_b_) of the strips were measured. Each test was performed in 6 replicates. Statistical analysis was performed with the Student *t*-test, with a *p* value of less than 0.05 considered to be significantly different.

Tungsten cumulative release from films was determined on portions of the films (2 × 2 cm), with each one placed into 2 mL of distilled water at room temperature. The solution was substituted at each time point (t = 3 h, 6 h, 1 d, 3 d, 7 d, and 14 d). The quantity of tungsten was measured by means of Agilent 4210 (Agilent, Santa Clara, CA, USA) molecular plasma-atomic emission spectroscopy (MP-AES). Tungsten lines at 400.871 and 429.461 nm were used [25]. The results from this analysis represent the mean value of three different determinations.

Film transmittance spectra were obtained in a Perkin Elmer Lambda45 spectrophotometer equipped with an integrating reflectance sphere (Labsphere RSA-PE-20, North Sutton, NH, USA). The spectra were recorded in the wavelength range of 270–900 nm with a scan rate of 480 nm/min.

### 4.4. Photochromic Properties

The photochromic properties of the gelatin films with WO_3_ nanoparticles (GW4, GW7, GTAW4, and GTAW7) were evaluated by performing transmittance spectra (wavelength range of 400–1100 nm, scan rate of 480 nm/min) of the films irradiated for 20 s by an LED at 365 nm at a distance of 3 cm, with an irradiance of 80 W/m^2^. Films without WO_3_ nanoparticles (GP4, GP7, GTAP4, and GTAP7) were also irradiated in the same conditions as the reference samples. The bleaching was also monitored at 60, 80, and 100 °C by placing the samples after irradiation in an oven for three days and monitoring them every day.

Irradiation cycles were performed by irradiating the films (GW4, GW7, GTAW4, and GTAW7) for ten seconds per cycle with the LED at 365 nm (distance of 3 cm and irradiance of 80 W/m^2^). After each irradiation cycle, transmittance spectra (wavelength range of 400–1100 nm, scan rate of 480 nm/min) were obtained. After two days of the irradiation cycle, transmittance spectra were obtained to verify bleaching due to the reverse photochromic reaction.

## Figures and Tables

**Table 1:** Film thickness, water vapor transmission rate (WVTR), and water vapor permeability (WVP).

Sample	Thickness × 10^−4^ (m)	WVTR (g h^−1^ m^−2^)	WVP × 10^−10^ (g s^−1^ m^−1^ Pa^−1^)	Sample	Thickness × 10^−4^ (m)	WVTR (g h^−1^ m^−2^)	WVP × 10^−10^ (g s^−1^ m^−1^ Pa^−1^)
G	1.13 ± 0.07	6.52 ± 0.26	1.23 ± 0.07	GTA	1.28 ± 0.06	4.49 ± 0.22	0.95 ± 0.05
GP4	1.86 ± 0.05 (*)	3.45 ± 0.07 (*)	1.07 ± 0.03	GTAP4	1.53 ± 0.07	4.32 ± 0.16	1.10 ± 0.04
GP7	1.77 ± 0.07 (*)	3.61 ± 0.09 (*)	1.06 ± 0.02	GTAP7	1.52 ± 0.07	4.40 ± 0.09	1.11 ± 0.04
GW4	1.80 ± 0.08 (*)	3.08 ± 0.25 (*)	0.92 ± 0.06 (*)	GTAW4	1.32 ± 0.05	4.40 ± 0.11	0.97 ± 0.02
GW7	1.87 ± 0.05 (*)	2.73 ± 1.22 (*)	0.85 ± 0.04 (*)	GTAW7	1.47 ± 0.08	3.82 ± 0.09	0.93 ± 0.02

* *p* < 0.01: thickness: GP4, GP7, GW4, and GW7 vs. G; WVTR: GP4, GP7, GW4, and GW7 vs. G; WVP: GW4 and GW7 vs. G.

**Table 2:** Values of water contact angle in degrees.

Sample	Contact Angle (°)	Sample	Contact Angle (°)
G	76.1 ± 1.4	GTA	78.3 ± 1.7
GP4	68.1 ± 0.8 (*)	GTAP4	65.6 ± 1.5 (***)
GP7	65.5 ± 1.3 (**)	GTAP7	65.1 ± 1 (***)
GW4	68.4 ± 1 (*)	GTAW4	67.2 ± 1.3 (***)
GW7	70.1 ± 1 (*)	GTAW7	69.2 ± 0.4 (***)

* *p* < 0.05: GP4, GW4, and GW7 vs. G; ** *p* < 0.01: GP7 vs. G; *** *p* < 0.01: GTAP4, GTAP7, GTAW4, and GTAW7 vs. GTA.

**Table 3:** Denaturation temperatures (T_D_) and enthalpies (ΔH_D_) recorded through DSC measurements.

Sample	T_D_ (°C)	ΔH_D_ (J/g)	Sample	T_D_ (°C)	ΔH_D_ (J/g)
G	93 ± 0.3	22.8 ± 0.4	GTA	95.3 ± 0.1	15.2 ± 0.4
GP4	91.6 ± 0.1	13.3 ± 0.2 (*)	GTAP4	95 ± 0.2	13.4 ± 0.1
GP7	90.8 ± 0.5	14.2 ± 0.3 (*)	GTAP7	94.6 ± 0.1	13.0 ± 0.4
GW4	92.1 ± 0.1	14.6 ± 0.2 (*)	GTAW4	94.7 ± 0.1	15.2 ± 0.1
GW7	90.4 ± 0.3	16.4 ± 0.2 (*)	GTAW7	94.3 ± 0.1	14.5 ± 0.4

* *p* < 0.01: GP4, GP7, GW4, and GW7 vs. G.

**Table 4:** Young’s modulus (E), stress at break (σ_b_), and strain at break (ε_b_) of gelatin films. Each value is the mean of at least six determinations and is reported with its standard deviation.

Sample	E (MPa)	σ_b_ (MPa)	ε_b_ (%)
G	2.0 ± 0.4	2.5 ± 0.3	152.3 ± 28
GP4	1.3 ± 0.3	2.1 ± 0.2	155.6 ± 39
GP7	1.3 ± 0.4	1.9 ± 0.3	147.9 ± 36
GW4	0.7 ± 0.08 (*)	1.4 ± 0.2 (*)	151.8 ± 29
GW7	0.7 ± 0.08 (*)	1.7 ± 0.2 (*)	152.3 ± 35

* *p* < 0.01: GW4, GW7 vs. G.

**Table 5:** Young’s modulus (E), stress at break (σ_b_), and strain at break (ε_b_) of crosslinked gelatin films. Each value is the mean of at least six determinations and is reported with its standard deviation.

Samples	E (MPa)	σ_b_ (MPa)	ε_b_ (%)
GTA	4.9 ± 0.6	3.1 ± 0.9	20 ± 0.9
GTAP4	4.1 ± 1	2.8 ± 0.3	17.4 ± 0.9
GTAP7	4.0 ± 0.9	3.0 ± 0.6	19.7 ± 1.2
GTAW4	3.3 ± 0.4 (*)	2.4 ± 0.9	20.9 ± 1.8
GTAW7	3.2 ± 0.2 (*)	2.5 ± 0.5	21.3 ± 1.7

* *p* < 0.05: GTAW4, GTAW7 vs. GTA.

## Data Availability

The data presented in this study are available on request from the corresponding author.

## Supplementary Materials

The following supporting information can be downloaded at https://www.mdpi.com/article/10.3390/gels10060354/s1, Figure S1: Photographs of gelatin films showing different colors due to WO_3_ nanoparticles and/or crosslinking with glutaraldehyde solution; Figure S2: FEG-SEM morphological investigation; Figure S3: EDS spectra of films; Figure S4: DSC thermograms; Figure S5: FT-IR spectra collected in ATR mode: comparison between GW7 and pure PVP and selected crosslinked films; Figure S6: Gelatin film photochromic effect. The left part of every sample was irradiated with an LED at 365 nm (irradiance 80 W/m^2^) for 20 s; Figure S7: Gelatin film photochromic effect efficiency. The irradiation was performed by an LED at 365 nm (irradiance 80 W/m^2^). The irradiation lasted until a transmittance value lower than 5% was achieved; Table S1: Main vibration modes and related wavenumbers in the FT-IR spectra of uncrosslinked films; Table S2: Main vibration modes and related wavenumbers in the FT-IR spectra of crosslinked films.
