# Supplementary material for: The Role of WO3 Nanoparticles on the Properties of Gelatin Films
Source: Gels. 2024 May 21;10(6):354. doi: 10.3390/gels10060354 (PMC11203329; doi:10.3390/gels10060354)
Supplement: Supplementary file 1 [file gels-10-00354-s001.zip › gels-3012618-supplementary.pdf]

Supplementary Materials

The role of WO_3 nanoparticles on the properties of gelatin films

Katia Rubini, Arianna Menichetti, Maria Cristina Cassani, Marco Montalti,

Adriana Bigi, Elisa Boanini

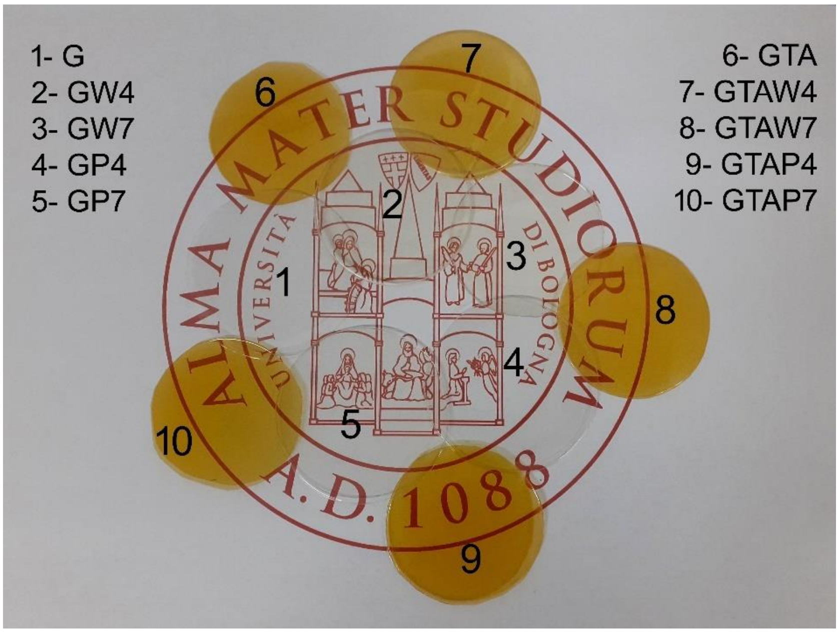

Figure S1 - Photographs of gelatin films showing the different colors due to WO_3 nanoparticles and/or crosslinking with glutaraldehyde solution.

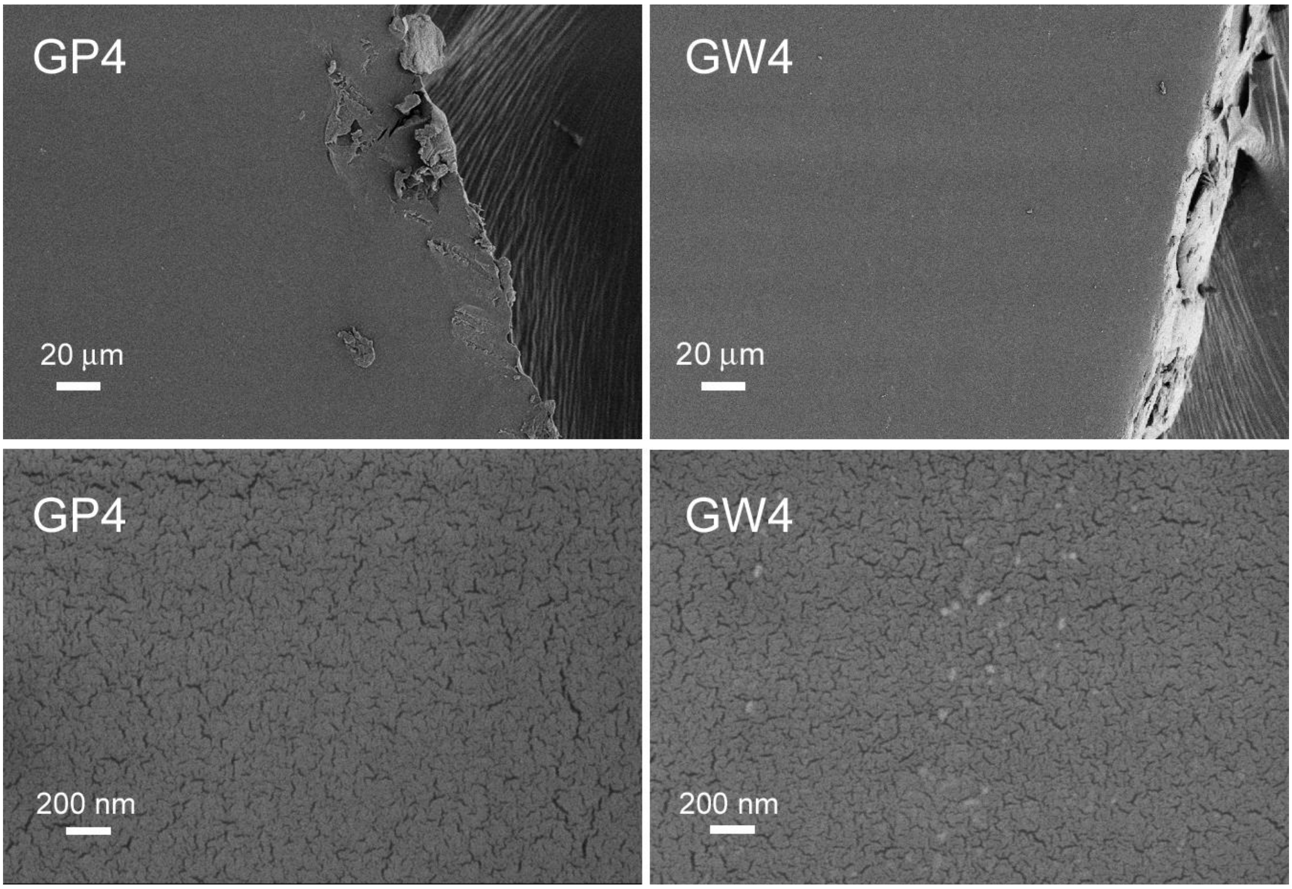

Figure S2 - FEG-SEM morphological investigation reveals the presence of aggregates of nanoparticles on the surfaces of gelatin films containing WO_3. The high-magnification images (100,000x) show the presence of sputtered gold.

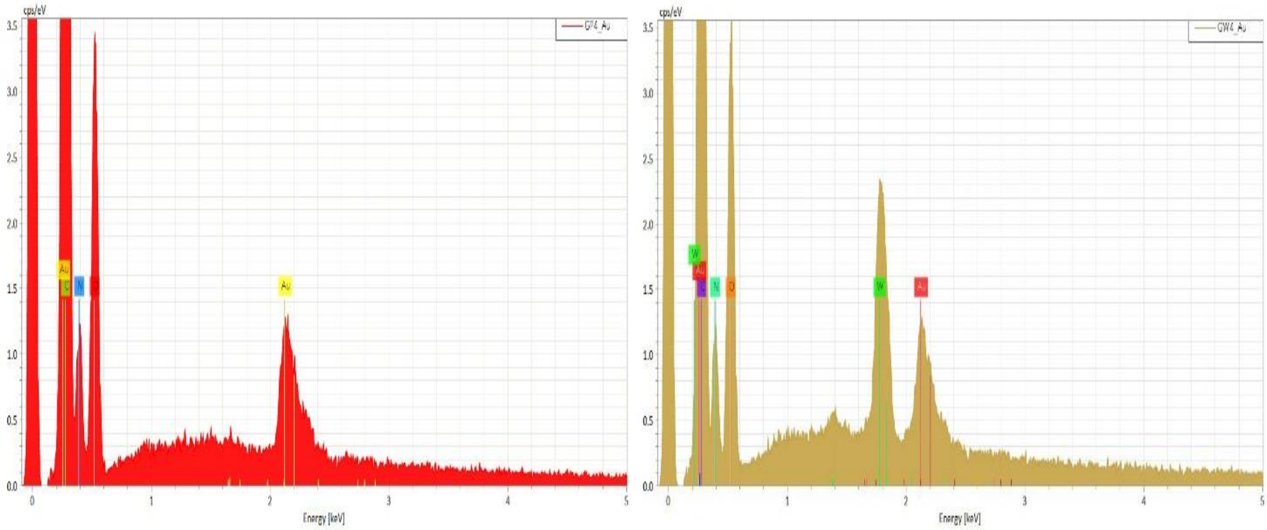

Figure S3 – EDS spectra of GW4 (WO_3 loaded, on the left) and GP4 (unloaded, on the right) films.

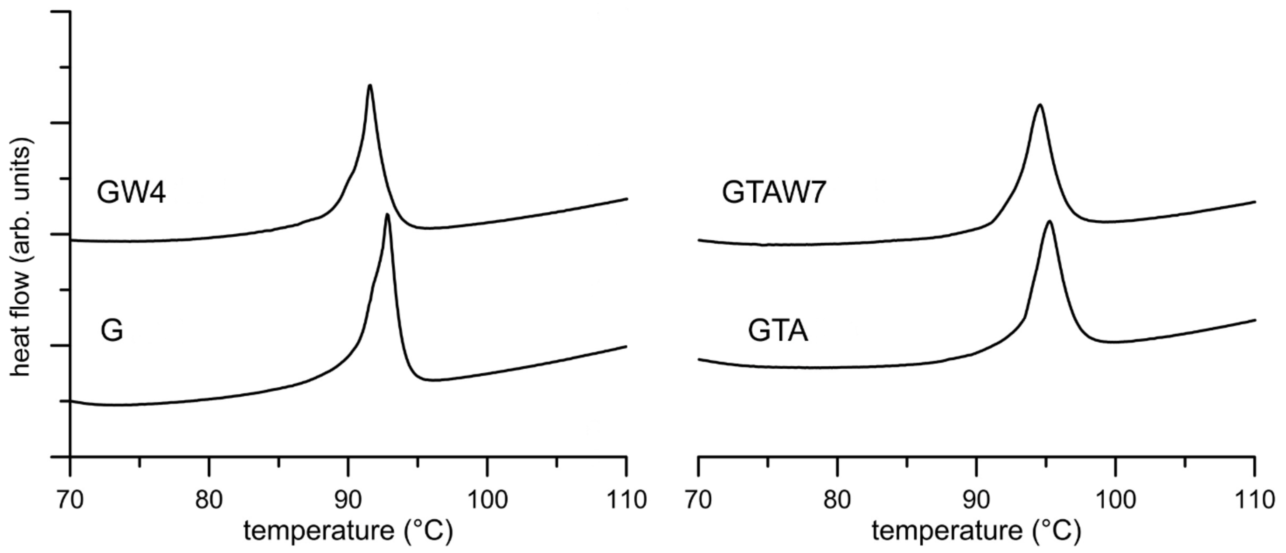

Figure S4 - DSC thermograms recorded from G films and GTA films show the presence of an endothermic peak due to collagen triple helix denaturation

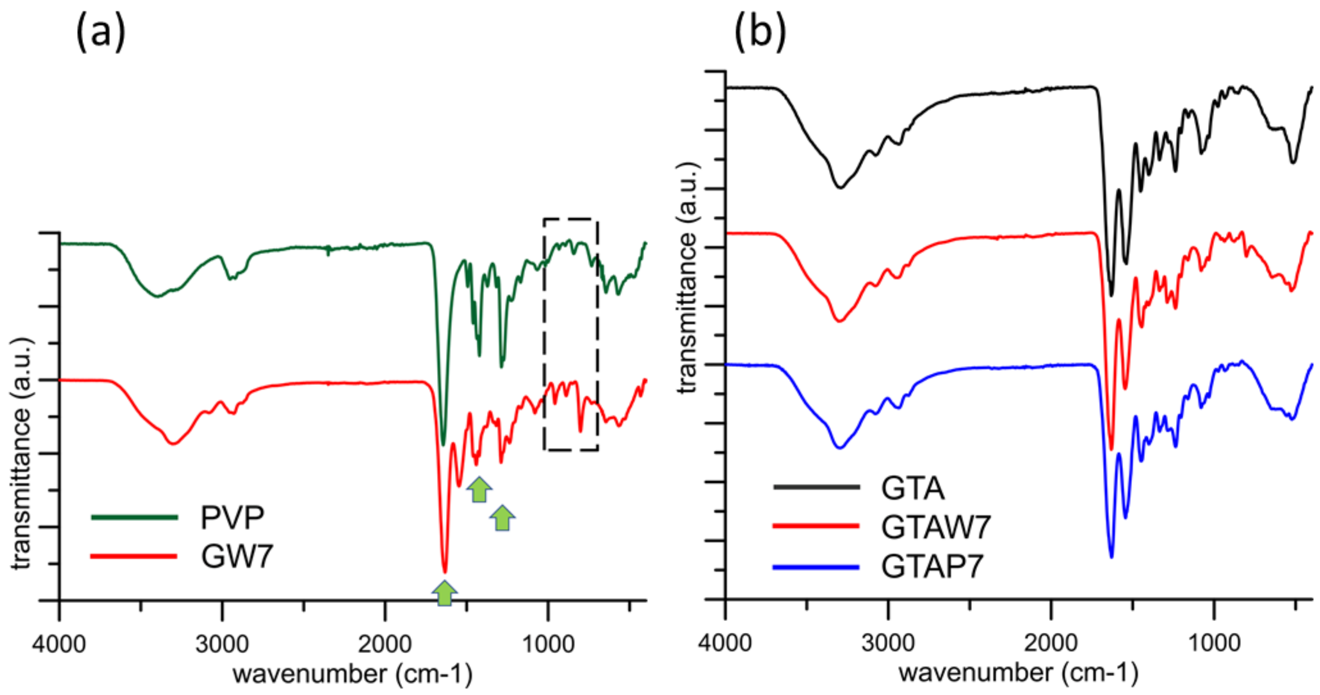

Figure S5 - FT-IR spectra collected in ATR mode: (a) comparison between GW7 and pure PVP; (b) selected crosslinked films. Green arrows indicate the absorption bands due to the presence of PVP ; dashed line indicates the absorption bands due to the nanoparticles.

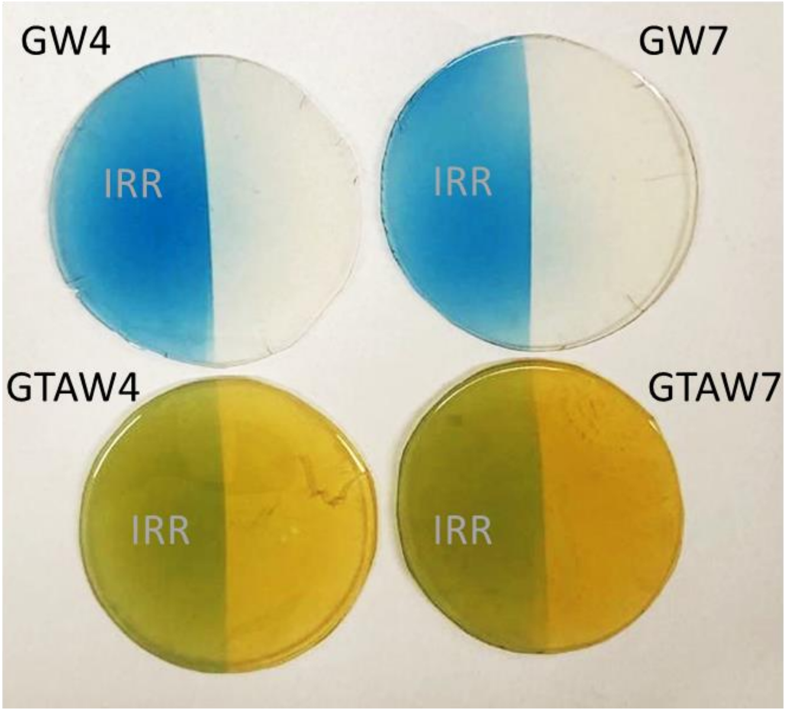

Figure S6 - Gelatin films photochromic effect. The left part of every sample has been irradiated with a LED at 365 nm (irradiance 80 W/m^2) for 20 seconds.

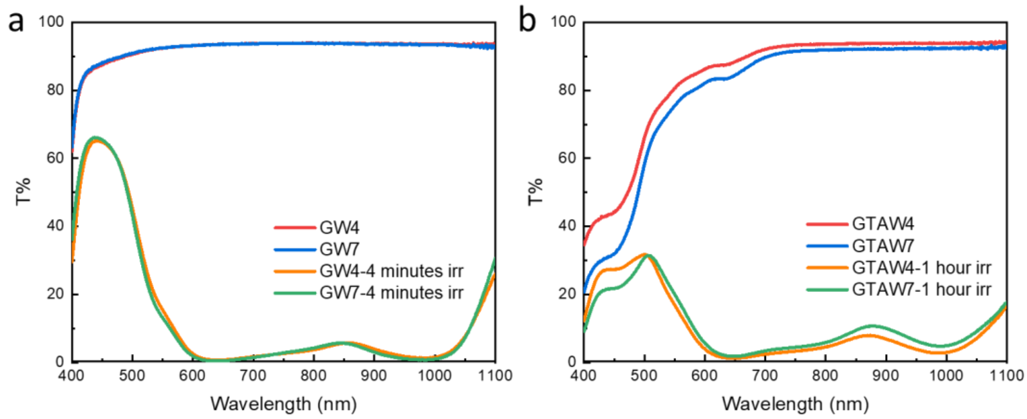

Figure S7 - Gelatin films photochromic effect efficiency. (a) GW4 and GW7, (b) GTAW4 and GTAW7. The irradiation was performed by a LED at 365 nm (irradiance 80 W/m^2). The irradiation lasted until a transmittance value lower than 5% was achieved.

Table S1 - Main vibration modes and related wavenumbers (cm^{-1}) in the FTIR spectra of non-crosslinked films. ν: stretching (s: symmetric, as: asymmetric), δ: bending

Vibration modes	**G**	**GW7**	**GP7**
ν-NH ; ν-OH	3450 - 3295	3485 - 3305	3490 – 3305
ν_s and νas -CH_2	2950 - 2880	2930 - 2875	2955-2880
ν C=O<br>*amide I*	1627	1632	1632
δ N-H ; ν C-N<br>*amide II*	1543	1550	1550
δ -CH_2	1450	1440	1440
δ -NH<br>*amide III*	1235	1289 - 1240	1283 - 1230
ν C-O	1080	1081	1081

Table S2 - Main vibration modes and related wavenumbers (cm^{-1}) in the FTIR spectra of crosslinked films. ν: stretching (s: symmetric, as: asymmetric), δ: bending

Vibration modes	**GTA**	**GTAW7**	**GTAP7**
ν-NH ; ν-OH	3510-3290	3570-3305	3485-3296
ν_s and ν_{as} -CH_2	3935-	2952-2878	2935-2875
ν C=O<br>*amide I*	1635	1633	1632
δ N-H ; ν C-N<br>*amide II*	1538	1547	1544
δ -CH_2	1452-1401	1444	1448-1400
δ -NH<br>*amide III*	1237	1288-1237	1284-1237
ν C-O	1078	1079	1080
